# Integrated bioinformatics analysis identifies hub genes and immune regulatory networks in HIV infection

**DOI:** 10.3389/fimmu.2025.1600713

**Published:** 2025-06-12

**Authors:** Xiaoxia Pang, Xinghong Chen, Yuxin Jing, Feng Shi, Xiaoying Chen, Huatuo Huang, Chunhong Liu

**Affiliations:** ^1^ Center for Medical Laboratory Science, Affiliated Hospital of Youjiang Medical University for Nationalities, Baise, China; ^2^ Key Laboratory of Research on Clinical Molecular Diagnosis for High Incidence Diseases in Western Guangxi of Guangxi Higher Education Institutions, Baise, China; ^3^ Key Laboratory of Research and Development on Clinical Molecular Diagnosis for High-Incidence Diseases of Baise, Baise, China; ^4^ Department of Medical Reproduction Center, Affiliated Hospital of Youjiang Medical University for Nationalities, Baise, China

**Keywords:** HIV infection, bioinformatics analysis, hub genes, immune, regulatory networks

## Abstract

**Introduction:**

Acquired Immune Deficiency Syndrome (AIDS) is a chronic and life-threatening condition caused by the human immunodeficiency virus (HIV), which severely weakens the immune system. Despite advances in treatment, AIDS remains incurable. Understanding the molecular mechanisms underlying AIDS progression is crucial for developing effective therapeutic strategies. Therefore, this study aims to identify hub genes associated with AIDS susceptibility and progression, as well as to elucidate potential molecular mechanisms involved.

**Methods:**

We used the Gene Expression Omnibus (GEO) dataset GSE76246 for this study. Differentially expressed genes (DEGs) were screened, and Weighted Gene Co-expression Network Analysis (WGCNA) was employed to construct gene modules associated with HIV infection. Hub genes were identified using the CytoHubba plugin, and their expression profiles were assessed using box plots. The diagnostic potential of these genes was evaluated using receiver operating characteristic (ROC) analysis. Functional enrichment and Gene Set Enrichment Analysis (GSEA) were conducted to identify key biological pathways. Additionally, we analyzed immune cell infiltration and constructed drug-gene interaction, miRNA and transcription factor (TF) regulatory networks.

**Results:**

101 intersection genes were identified by combining DEGs, Oxidative stress genes and module genes from WGCNA. Functional enrichment analysis highlighted key pathways, including oxidative stress response and apoptotic signaling. A protein-protein interaction (PPI) network analysis identified 10 hub genes (TP53, AKT1, JUN, CTNNB1, PXDN, MAPK3, FOS, MMP9, FOXO1, STAT1), which showed strong diagnostic potential, as evidenced by ROC curve analysis. Immune infiltration analysis revealed significant associations between hub genes and various immune cell populations. Furthermore, drug-gene interaction analysis predicted several potential therapeutic compounds. Additionally, miRNA and TF regulatory networks were constructed, identifying critical regulatory elements influencing the expression of hub genes.

**Conclusion:**

This study identified ten hub genes (TP53, AKT1, JUN, CTNNB1, PXDN, MAPK3, FOS, MMP9, FOXO1, STAT1) that play crucial roles in HIV infection and progression. These genes serve as potential biomarkers for HIV diagnosis and therapeutic targets.

## 1 Introduction

Human immunodeficiency virus (HIV) infection remains a severe global public health issue, imposing significant health and economic burdens worldwide. According to the Global HIV/AIDS Statistics for 2023, approximately 39.9 million individuals are living with HIV. Among them, 30.7 million were receiving antiretroviral therapy (ART), 1.3 million were newly infected, and 630,000 died from AIDS-related illnesses (1). One of the indications of infection is the continuous destruction of CD4+ T cells, which eventually collapses the human immune system leading to the acquired immune deficiency syndrome (AIDS) and increased mortality (2). Although ART effectively suppresses viral replication and reduces transmission, HIV/AIDS remains incurable because the virus integrates into the chromosomes of latent reservoirs and persists for life (3). At the molecular level, HIV infection not only induces interactions between viral and host genes but also involves complex regulatory networks of host genes. In recent years, approaches such as gene expression profiling, genome-wide association studies (GWAS), and transcriptomics analyses have been widely employed to identify genes associated with HIV infection. Previous studies have uncovered several key genes involved in HIV susceptibility, disease progression, and immune responses (4–8). However, the regulatory mechanisms and network characteristics of these genes remain largely unexplored.

It is well established that the immune system and immune cells play a central role in the pathogenesis of HIV infection (9, 10). The immune system consists of innate and adaptive immune cells, including T cells, B cells, dendritic cells (DCs), macrophages, and neutrophils, which mediate both active and suppressive immune functions (11). HIV infection profoundly alters immune homeostasis, disrupting both innate and adaptive immune responses. The virus directly targets CD4+ T cells, leading to their depletion and dysfunction, and disrupts the activity of other immune cells. This immune dysregulation is mediated through complex gene regulatory mechanisms, including alterations in transcriptional activity, post-transcriptional modifications, and signaling pathways involved in immune activation, apoptosis, and oxidative stress primarily p53 signaling pathway, NF-κB signaling pathway, and JAK-STAT signaling pathway (12–14). Key genes such as TP53, AKT1, STAT1, and FOXO1 have been implicated in the regulation of immune responses to HIV, particularly through pathways governing apoptosis, oxidative stress, and inflammatory signaling. These findings underscore the importance of elucidating gene regulatory networks in the context of HIV infection, which may reveal novel therapeutic targets and biomarkers.

Bioinformatic analysis can process large amounts of samples within an extremely short time and provide valuable information about diseases, which facilitates the identification of the gene regulatory networks, key genes and their associated pathways in various diseases. Such approaches have been widely applied in the study of multiple diseases (15–17) and they are equally relevant to HIV infection. To further elucidate the key genes involved in HIV infection and their potential molecular mechanisms, this study employed a combined approach, utilizing differential expression gene (DEG) screening and weighted gene co-expression network analysis (WGCNA). These techniques were used to construct gene network modules that are closely associated with HIV infection. Additionally, protein-protein interaction (PPI) networks and functional enrichment analyses (GO and KEGG) were employed to identify potential signaling pathways. Furthermore, by analyzing the regulatory networks of key genes, including microRNA and transcription factor (TF) regulation, along with performing immunoinfiltration analysis, this study explored the roles of these genes in immune regulation and their relationships with immune cell subpopulations, thereby uncovering the regulatory mechanisms of HIV infection at both the molecular and cellular levels.

Thus, the aim of this study is to elucidate the potential molecular mechanisms of immune-related genes as biomarkers for HIV infection through multiple bioinformatic approaches, offering novel insights into the diagnosis and treatment of HIV infection.

## 2 Methods

### 2.1 Data collection and study design

**Figure 1:**
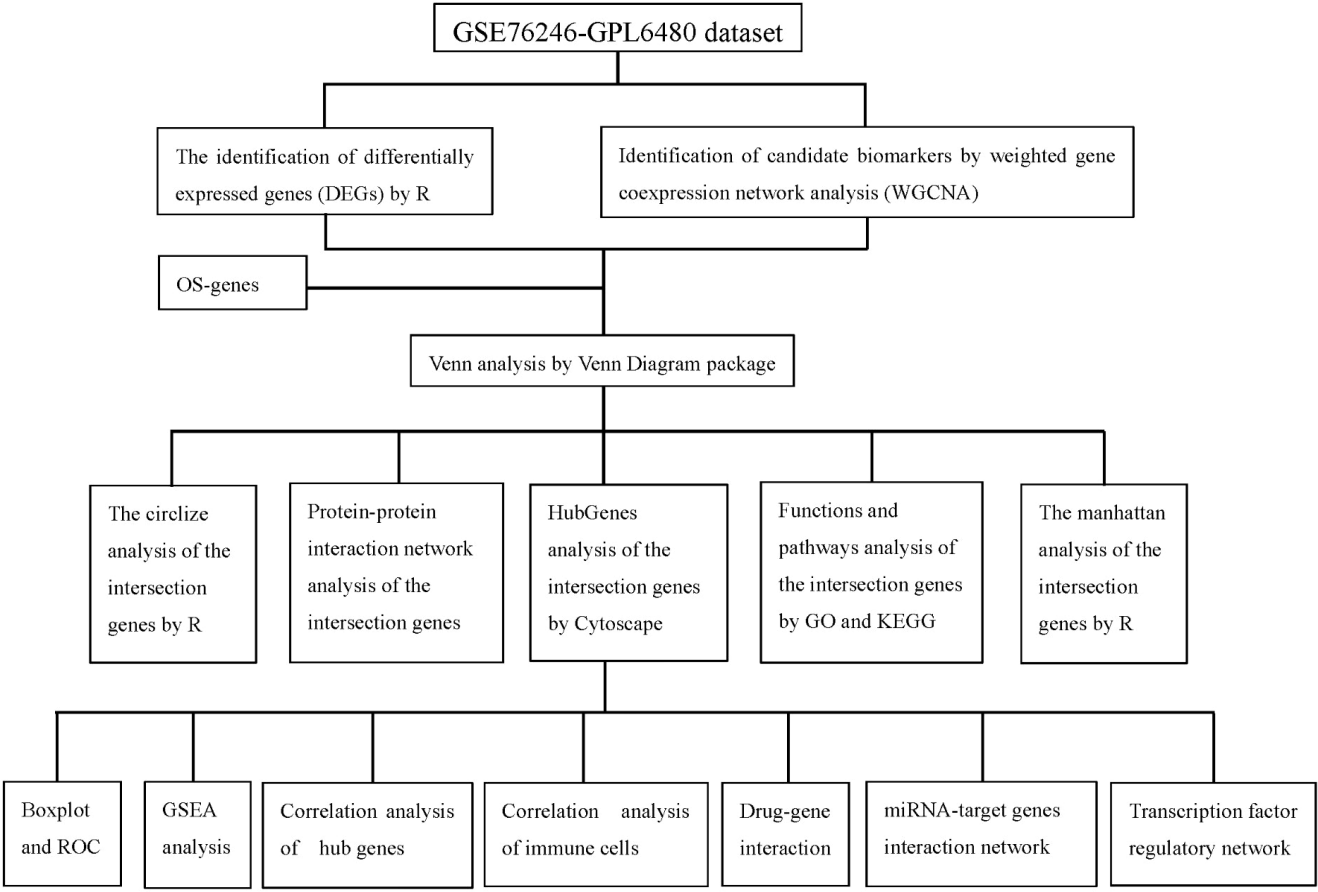
Flowchart of bioinformatic analysis.

In this study, the Gene Expression Omnibus (GEO) database (https://www.ncbi.nlm.nih.gov/geo/) was accessed, and case-control experimental data on humans within the past five years were screened using “HIV” as the keyword. The GSE76246 dataset (platform: GPL6480) was ultimately selected for analysis. This dataset includes gene expression profiles from peripheral blood mononuclear cells (PBMCs) of 30 patients in the chronic phase of HIV infection and 7 healthy controls. oxidative stress-related genes (OSRGs) were obtained from the Gene Ontology (GO) database (Data cohort: GO:0006979). The detailed technical workflow and bioinformatics analysis pipeline of this study are shown in **Figure 1**.

### 2.2 Identification of DEGs

The gene expression matrix of 37 samples was obtained by normalizing the expression data from the GSE76246 dataset. DEGs between 30 HIV-positive and 7 HIV-negative samples were identified using the R package limma. Specifically, DEGs were defined as those with a p-value threshold of < 0.05 and |log Fold change (FC)| > 0.5.

### 2.3 Weighted gene co-expression network analysis

We constructed the gene co-expression network using the expression profile data of all genes from the GSE76246 dataset and identified key modules using the WGCNA package in R. Gene modules and clinical phenotypes (control and HIV-positive) were correlated using Pearson’s correlation coefficient. For the HIV-positive group, gene modules with a higher correlation coefficient and lower p-value were selected, and the genes associated with these modules were further analyzed.

### 2.4 Identification of intersection genes

To identify the intersection genes, Venn analysis was used to combine DEGs, Oxidative stress genes, and module genes identified through WGCNA using the Venn diagram package. This approach enabled the identification of intersection genes that contribute to the pathogenesis of HIV infection.

### 2.5 Enrichment analysis using GO and KEGG pathway analysis

To identify the biological functions and signaling pathways of genes, we performed Gene Ontology (GO) and Kyoto Encyclopedia of Genes and Genomes (KEGG) pathway enrichment analysis using the “clusterProfiler” package. We examined three categories: biological process (BP), cellular component (CC), and molecular function (MF) to explore the biological functions of the intersection genes. Additionally, signaling pathways related to HIV-positive status were investigated through KEGG analysis.

### 2.6 Protein–Protein interaction (PPI) network construction and screening of hub genes

The PPI network of the intersection genes was retrieved from the STRING online database (https://cn.string-db.org/) (18). The constructed PPI network was then imported into Cytoscape software for visualization to identify potential relationships. To screen for hub genes, we used CytoHubba, a Cytoscape plug-in, to select the top 10 ranked genes from the 101 intersection genes based on the degree method as hub genes.

### 2.7 The expression and diagnostic performance of hub genes

The expression of the hub genes was exhibited by box plots. The ROC was used to evaluate the diagnostic performance of the hub genes for identifying HIV-positive status.

### 2.8 Implementation of GSEA for single hub genes

After obtaining the hub genes, we further performed the GSEA analysis of these genes by GSEA-KEGG function in clusterProfiler package. The adjusted p-value < 0.05 was set as the cut-off criteria.

### 2.9 Construction of drug-gene interaction network, miRNA network, and transcriptional factor regulatory network

The public online platforms, including https://dgidb.genome.wustl.edu/, https://www.mirnet.ca/, and https://www.mirnet.ca/upload/GeneUploadView.xhtml, were used to study drug-gene interactions, miRNA networks, and TF regulation, respectively. The networks were then created and visualized using Cytoscape.

### 2.10 Statistical analysis

All analyses in our current study were conducted with R software (Version 4.3.1) and its several open packages. *P* < 0.05 was considered statistically significant.

## 3 Results

### 3.1 Identification of DEGs in the HIV-positive patients

**Figure 2:**
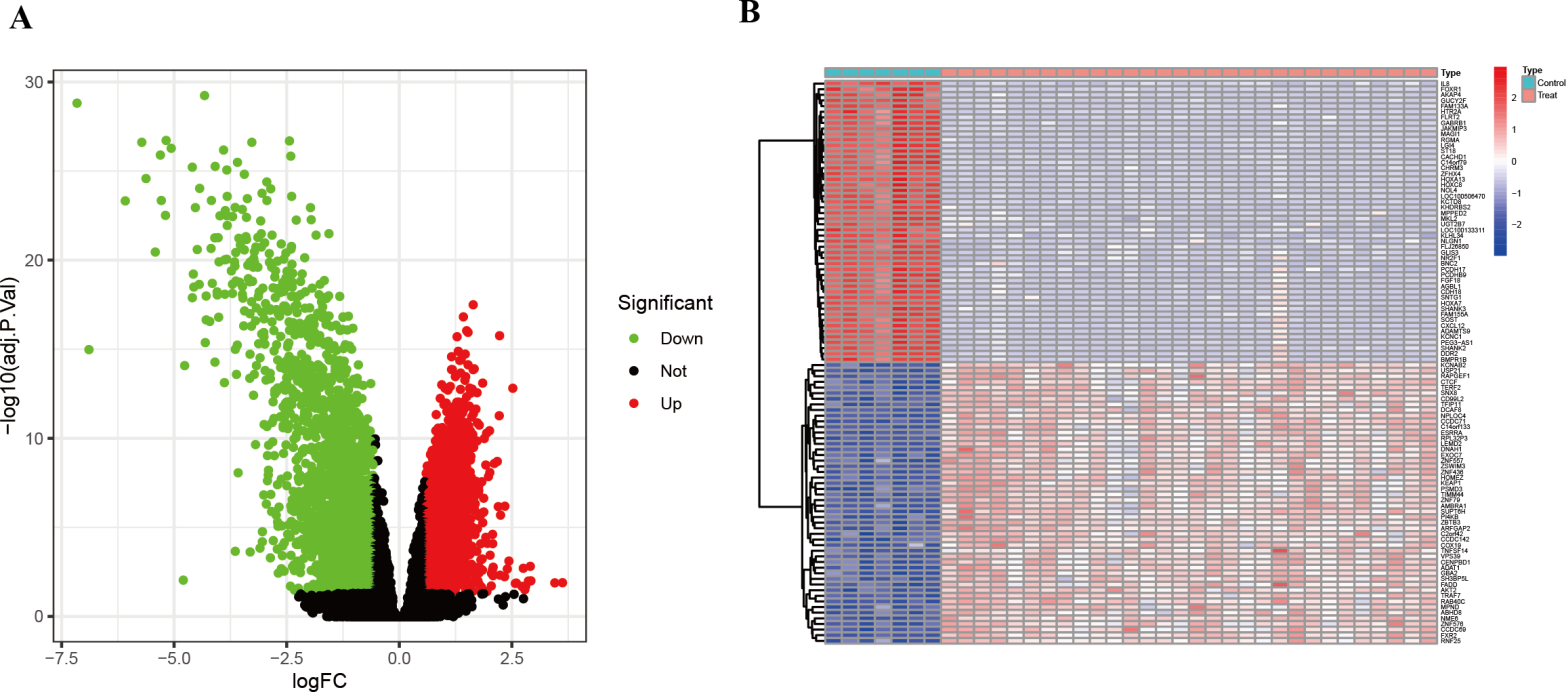
DEGs between HIV-positive patients and controls. **(A)** Volcano plot showed all DEGs of HIV-positive patients in contrast to controls. **(B)** The heatmap illustrates the top 50 up-regulated and 50 down-regulated DEGs in HIV-positive patients.

To identify genes differentially expressed between HIV-positive patients and controls, a volcano plot was generated, revealing up-regulated genes in red and down-regulated genes in green, with non-significant genes shown in black (**Figure 2A**). A heatmap further illustrated the top 50 up-regulated and 50 down-regulated DEGs (**Figure 2B**). These DEGs served as a foundation for subsequent co-expression analysis.

### 3.2 WGCNA and identification of module associated with HIV-positive patients

**Figure 3:**
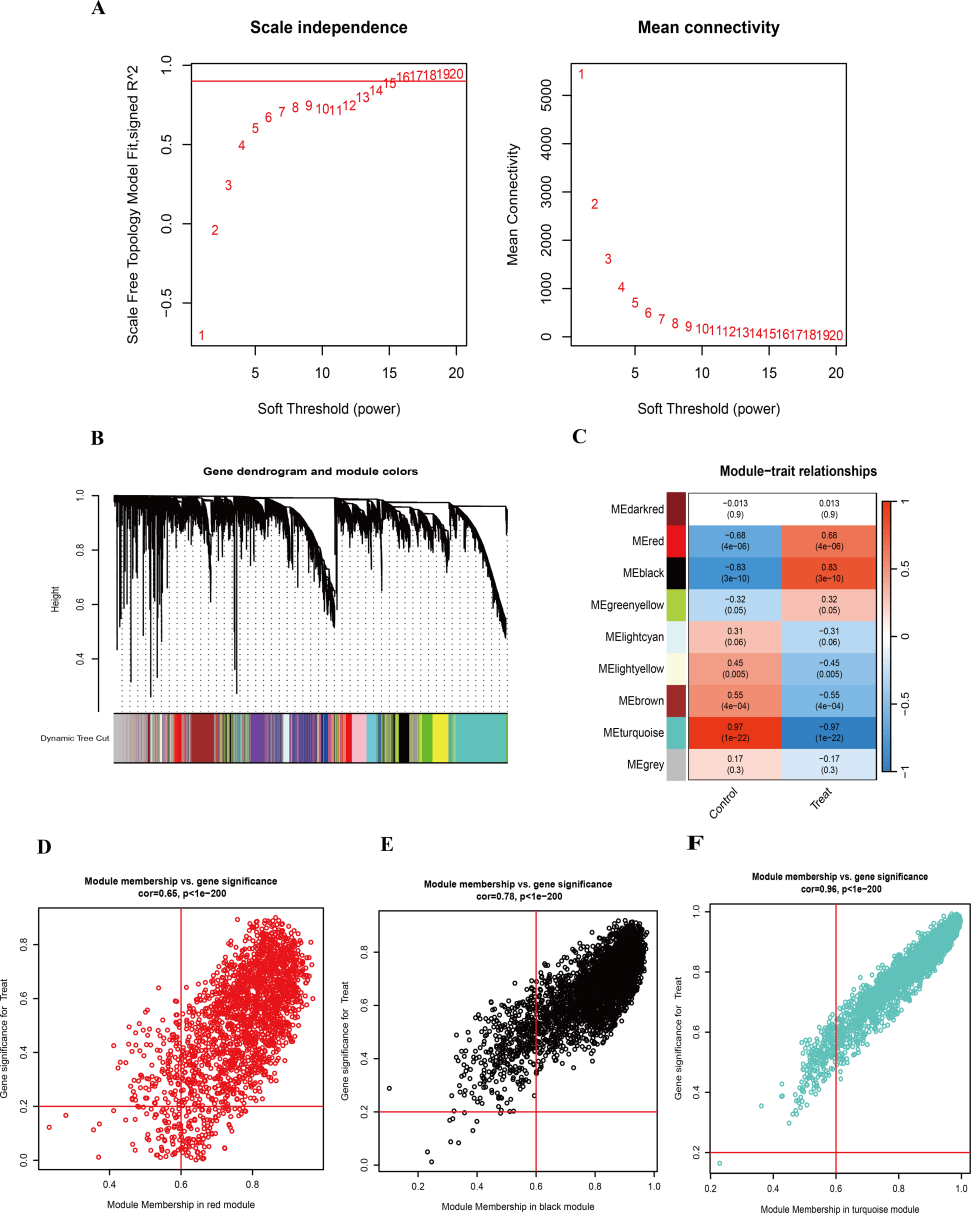
WGCNA and identification of highly related modules. **(A)** Determination of the soft-threshold power for HIV-positive samples. **(B)** Hierarchical clustering dendrogram of highly connected genes in key modules associated with HIV-positive status. **(C)** Correlations between gene modules and clinical traits in HIV-positive samples, with corresponding correlation coefficients and *P*-values displayed in each cell. **(D)** Scatter plot of module eigengenes associated with HIV-positive status in the red module. **(E)** Scatter plot of module eigengenes associated with HIV-positive status in the black module. **(F)** Scatter plot of module eigengenes associated with HIV-positive status in the turquoise module.

To investigate the correlation between genes and HIV-positive status, we performed WGCNA in addition to analyzing the differential expression between the two groups. A co-expression network was constructed using the soft-thresholding approach. Notably, the parameter β is critical for co-expression networks, as it affects both the average connectivity degree and the independence of co-expression modules. As shown in **Figure 3A**, β=16 was considered in this study. The hierarchical clustering dendrogram of the analysis is presented in **Figure 3B**. Furthermore, hierarchical clustering resulted in nine distinct gene modules, identified using the dynamic tree cut method. As displayed in **Figure 3C**, the association between each module and HIV-positive status was calculated. The results indicated that five modules were significantly correlated with HIV-positive status: the MEred module (cor = 0.68, *P* < 0.05), the MEblack module (cor = 0.83, *P* < 0.05), the MElightyellow module (cor = -0.45, *P* < 0.05), the MEbrown module (cor = -0.55, *P* < 0.05), and the MEturquoise module (cor = -0.97, *P* < 0.05). To refine the selection of candidate genes, we focused on modules with correlation values greater than 0.6 for subsequent analysis. Scatter plots of module eigengenes associated with HIV-positive status in the turquoise, black, and red modules were presented in **Figures 3D–F**.

### 3.3 Identification of intersection genes

**Figure 4:**
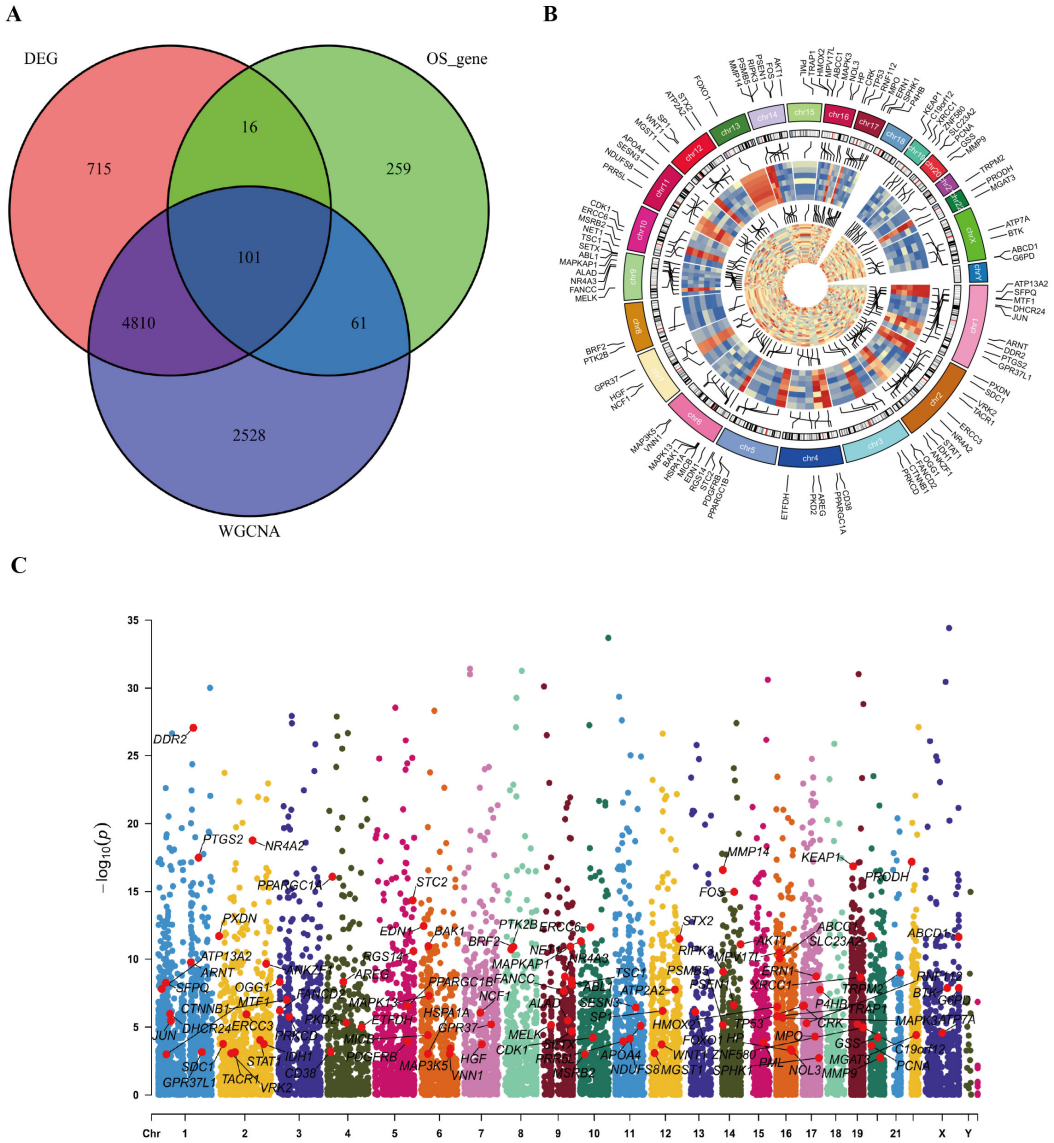
Identification of intersection genes and their positions on chromosome. **(A)** The Venn diagram for intersection genes. **(B)** positions of intersection genes on chromosome presented by ring heat map. **(C)** positions of intersection genes on chromosome presented by Manhattan diagram.

To refine the candidate gene set, we identified the overlap between DEGs and genes within the three most HIV-related WGCNA modules, yielding 4,911 intersection genes (**Figure 4A**). By further intersecting these with known oxidative stress-related genes, 101 genes were identified. Their chromosomal positions were visualized with a ring heatmap and a Manhattan plot (**Figures 4B, C**), laying the groundwork for functional enrichment analysis.

### 3.4 GO enrichment analysis and KEGG pathway analysis of the intersection genes

**Figure 5:**
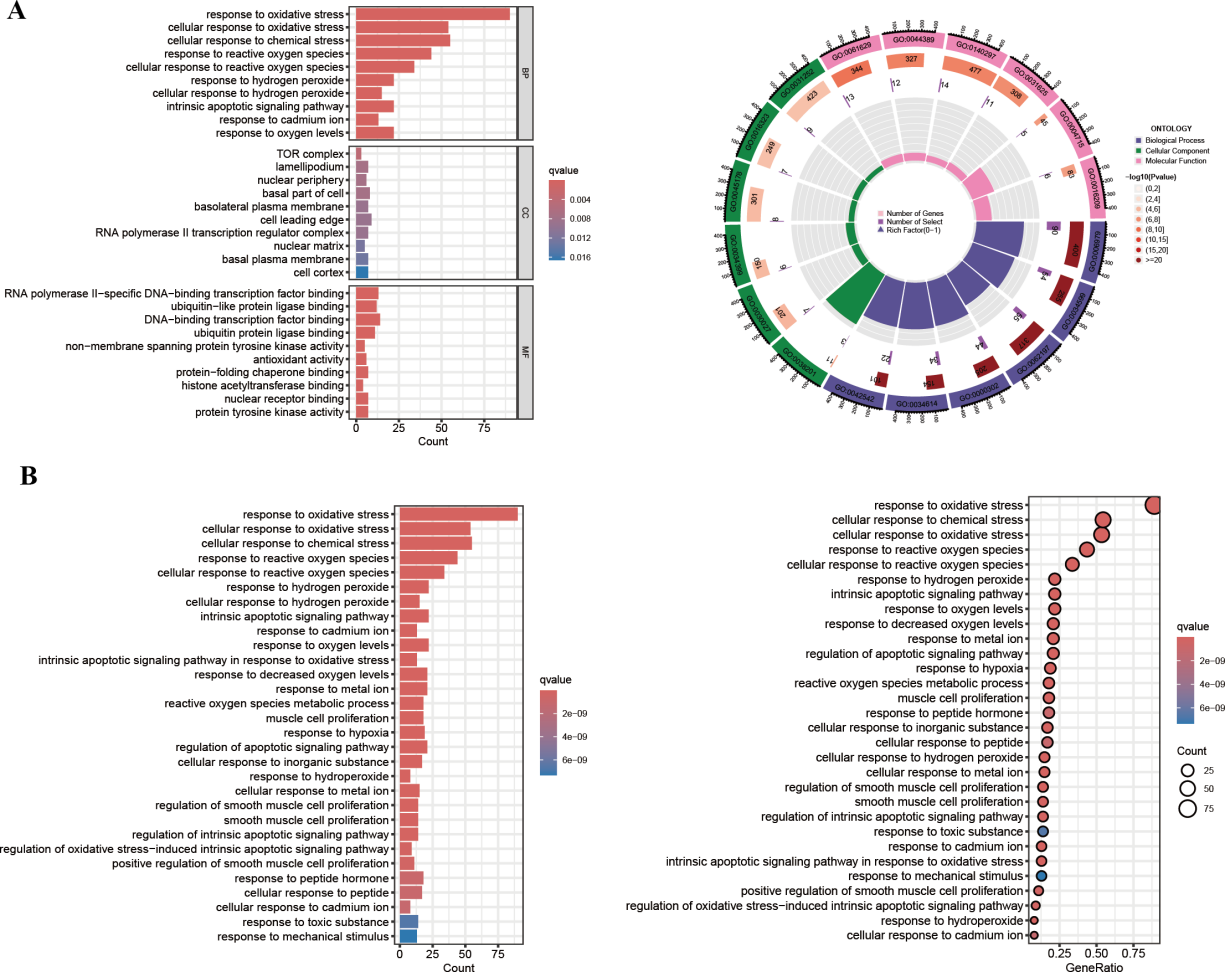
GO enrichment and KEGG pathway analysis. **(A)** The GO circle plot illustrates the scatter map of the logFC of the intersection genes. **(B)** The KEGG bar plot and bubble plot illustrate the scatter map of the logFC of the intersection genes.

We next sought to elucidate the biological roles of the 101 intersection genes through GO and KEGG enrichment analyses. The GO enrichment analysis consisted of three categories: BP, CC, and MF. Notably, functional enrichment was primarily focused on BP (**Figure 5A**). As shown in **Figure 5A**, the top three enriched pathways in the BP analysis were response to oxidative stress, cellular response to oxidative stress, and cellular response to chemical stress. In the CC analysis, the top three locations were the TOR complex, lamellipodium, and nuclear periphery. Additionally, in the MF analysis, RNA polymerase II−specific DNA−binding transcription factor binding, ubiquitin−like protein ligase binding, and DNA−binding transcription factor binding were identified as significant. Furthermore, the KEGG enrichment results were consistent with the GO analysis (**Figure 5B**). Notably, several apoptotic signaling pathways, likely related to the infectious process of HIV, were enriched, including the intrinsic apoptotic signaling pathway, regulation of apoptotic signaling pathway, regulation of intrinsic apoptotic signaling pathway, and regulation of oxidative stress−induced intrinsic apoptotic signaling pathway.

### 3.5 Intersection genes PPI network and screening of hub genes

**Figure 6:**
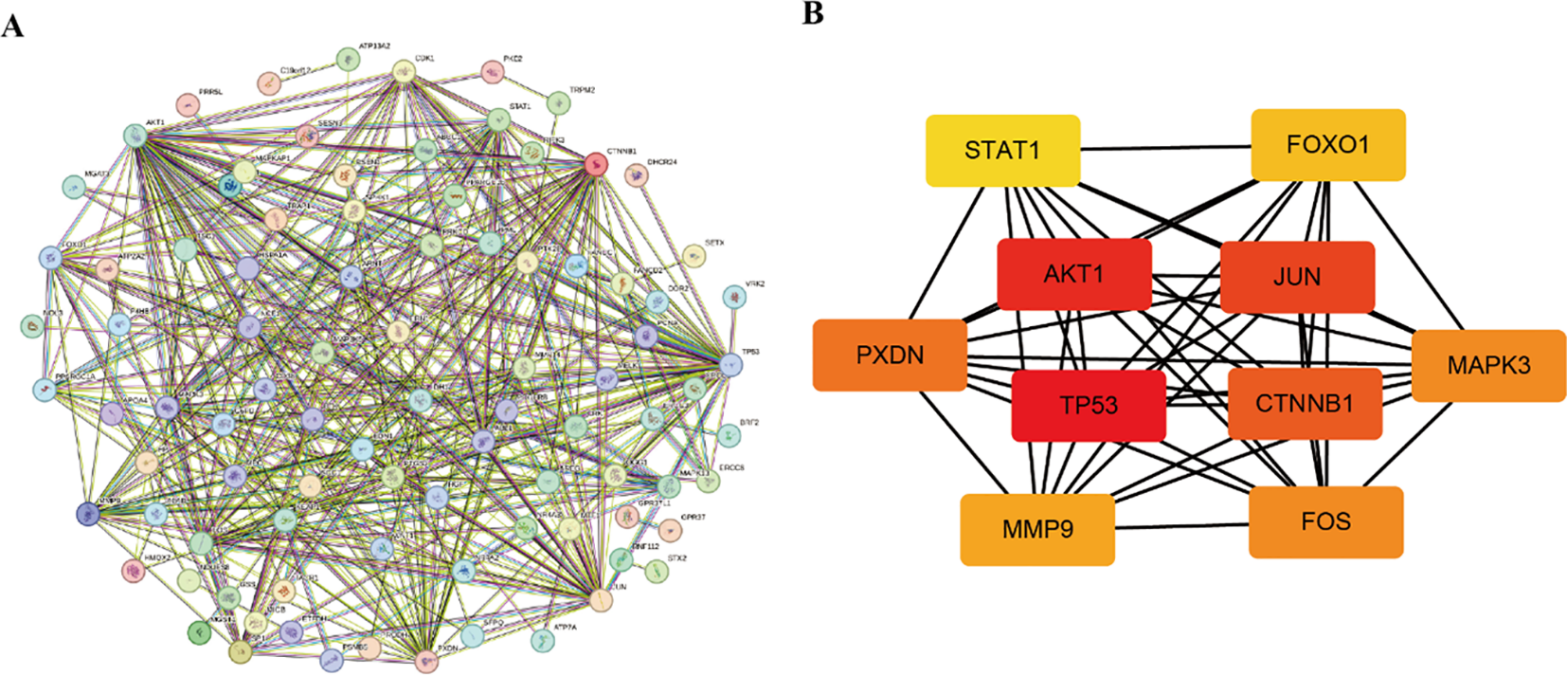
PPI network. **(A)** Construction of the intersection gene network. **(B)** Identification of the hub genes.

To determine key regulatory genes, we constructed a PPI network based on the intersection genes. A total of 101 intersection genes were imported into the PPI network complex, and free genes were removed. Finally, 434 pairs of interacting proteins were obtained in our study (**Figure 6A**). The interaction relationship was represented by nodes and edges, with combined scores used to indicate the strength of the interaction between two proteins. The higher the score, the closer the relationship. To screen the hub genes, these 101 intersection genes were ranked using the degree method in Cytoscape software. Genes with the top 10 highest scores were selected as hub genes (TP53, AKT1, JUN, CTNNB1, PXDN, MAPK3, FOS, MMP9, FOXO1, STAT1) (**Figure 6B**), and their specific scores are shown in **Table 1**. These hub genes were subjected to further expression and diagnostic performance analyses.

**Table 1:** The specific scores of hub genes.

Rank	Name	Score
1	TP53	59
2	AKT1	44
3	JUN	40
4	CTNNB1	36
5	PXDN	30
6	MAPK3	27
6	FOS	27
8	MMP9	25
9	FOXO1	22
10	STAT1	21

### 3.6 Expression level and diagnostic performance of hub genes

**Figure 7:**
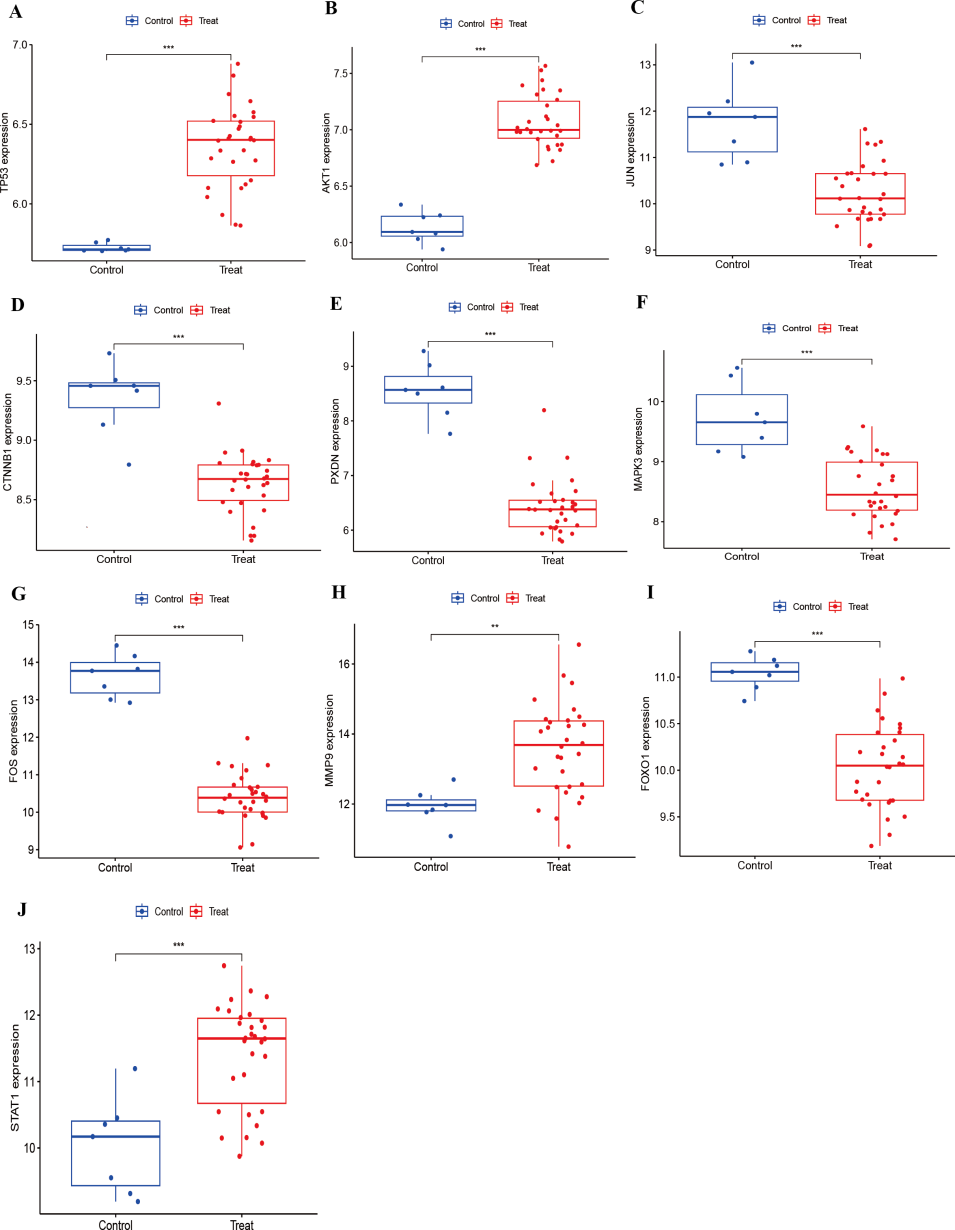
Expression of hub genes in HIV-Positive patients and controls. **(A–J)** show the expression levels of TP53, AKT1, JUN, CTNNB1, PXDN, MAPK3, FOS, MMP9, FOXO1, and STAT1, respectively. **p < 0.01, ***p < 0.001.

**Figure 8:**
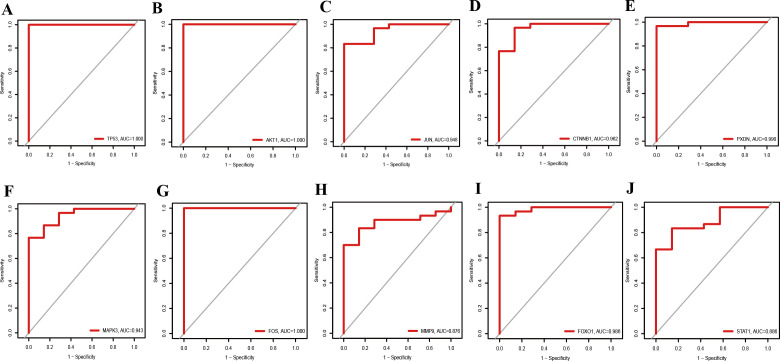
ROC curves for diagnostic performance of hub genes in HIV-Positive patients. **(A–J)** show the ROC curves for TP53, AKT1, JUN, CTNNB1, PXDN, MAPK3, FOS, MMP9, FOXO1, and STAT1, respectively.

**Figure 9:**
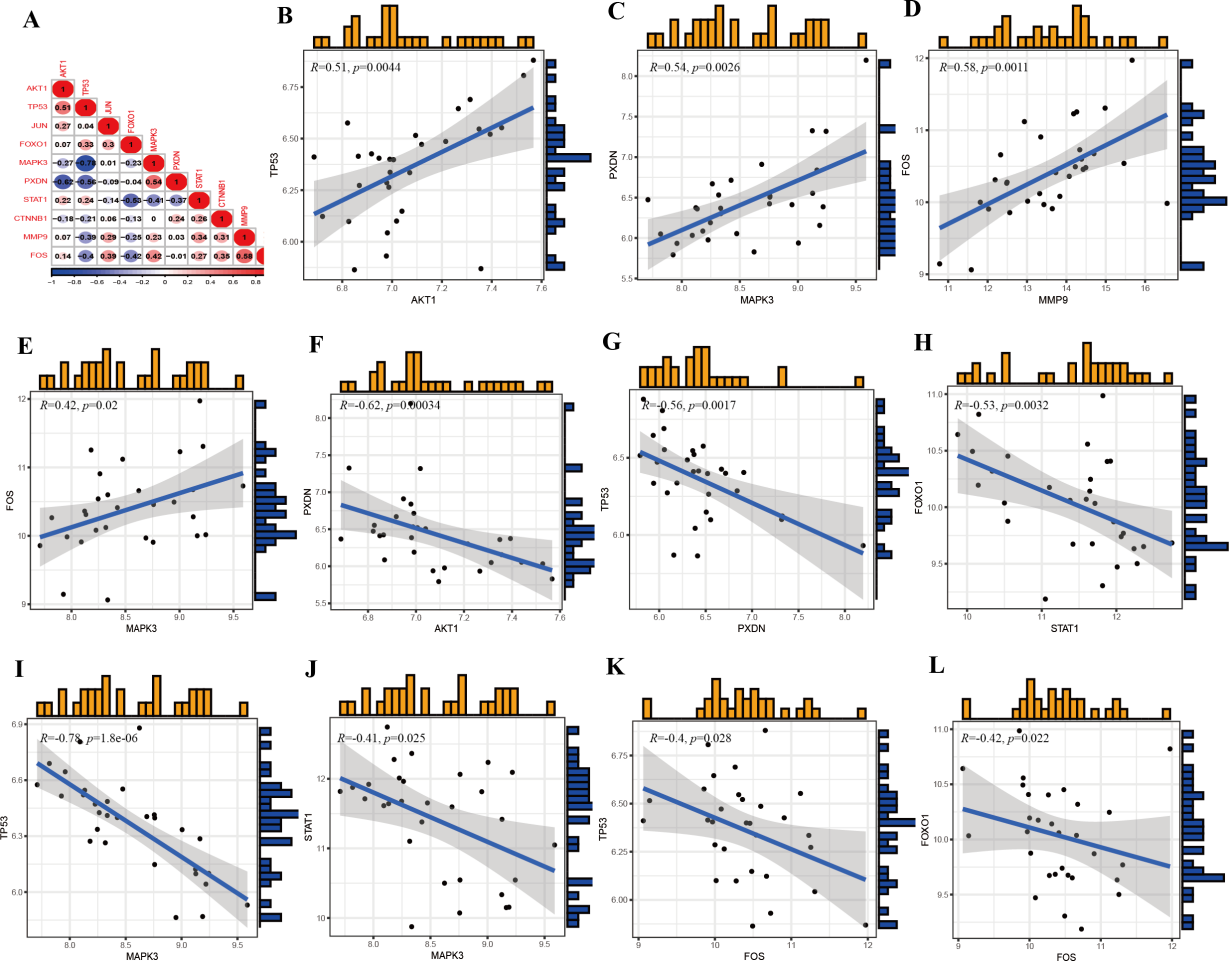
Correlations of the hub genes. **(A)** Correlation matrix of ten hub genes. **(B)** Correlation between TP53 and AKT1. **(C)** Correlation between PXDN and MAPK3. **(D)** Correlation between FOS and MMP9. **(E)** Correlation between FOS and MAPK3. **(F)** Correlation between PXDN and AKT1. **(G)** Correlation between TP53 and PXDN. **(H)** Correlation between FOXO1 and STAT1. **(I)** Correlation between TP53 and MAPK3. **(J)** Correlation between STAT1 and MAPK3. **(K)** Correlation between TP53 and FOS. **(L)** Correlation between FOXO1 and FOS.

The prediction and the discriminatory ability of the hub genes were assessed by analyzing the expression levels between HIV-positive patients and controls. Among these ten hub genes, TP53, AKT1, MMP9, and STAT1 were upregulated (**Figures 7A, B, H, J**), while JUN, CTNNB1, PXDN, MAPK3, FOX and FOXO1 were downregulated (**Figures 7C-G, I**). For a more precise understanding of diagnostic value for HIV-positive patients, the specificity and sensitivity of the hub genes were assessed using ROC curve analysis. The areas under the ROC curve (AUC) for each gene were as follows: 1.000 for TP53, 1.000 for AKT1, 0.948 for JUN, 0.962 for CTNNB1, 0.990 for PXDN, 0.943 for MAPK3, 1.000 for FOS, 0.876 for MMP9, 0.986 for FOXO1 and 0.886 for STAT1 (**Figures 8A-J**). These results indicate that these ten genes have a prominent capacity to distinguish between HIV-positive and HIV-negative individuals. Furthermore, the correlations between these ten hub genes were analyzed (**Figure 9**). **Figures 9B-E** show positive correlations between pairs of genes, while **Figures 9F-L** show negative correlations (P < 0.05). These findings demonstrate that the identified hub genes exhibit strong diagnostic potential and distinct expression patterns, effectively distinguishing HIV-positive from HIV-negative individuals.

### 3.7 KEGG pathway enrichment re-analysis of 10 hub genes

**Figure 10:**
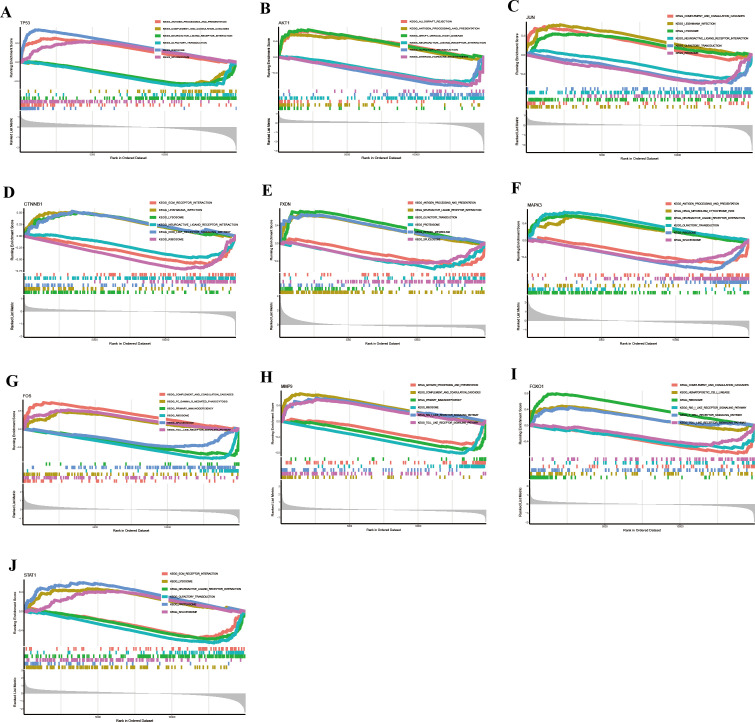
KEGG pathway analysis of hub genes via GSEA identified signaling pathways associated with HIV-infected patients. **(A)** TP53. **(B)** AKT1. **(C)** JUN. **(D)** CTNNB1. **(E)** PXDN. **(F)** MAPK3. **(G)** FOS. **(H)** MMP9. **(I)** FOXO1. **(J)** STAT1.

Subsequently, we performed single-gene GSEA analysis of the hub genes, and the top three upregulated and downregulated pathways were visualized using the “clusterProfiler” package. KEGG pathway analysis revealed the following associations:

TP53 was linked to antigen processing and presentation, complement and coagulation cascades, neuroactive ligand-receptor interaction, olfactory transduction, ribosome, and spliceosome (**Figure 10A**).

AKT1 was involved in allograft rejection, antigen processing and presentation, graft-versus-host disease, neuroactive ligand-receptor interaction, olfactory transduction, and steroid hormone biosynthesis (**Figure 10B**).

JUN was associated with complement and coagulation cascades, leishmaniasis infection, lysosome, neuroactive ligand-receptor interaction, olfactory transduction, and ribosome (**Figure 10C**).

CTNNB1 participated in ECM-receptor interaction, leishmaniasis infection, lysosome, neuroactive ligand-receptor interaction, NOD-like receptor signaling pathway, and ribosome (**Figure 10D**).

PXDN was involved in antigen processing and presentation, complement and coagulation cascades, olfactory transduction, proteasome, retinol metabolism, and spliceosome (**Figure 10E**).

MAPK3 took part in antigen processing and presentation, drug metabolism (cytochrome P450), neuroactive ligand-receptor interaction, olfactory transduction, ribosome, and spliceosome (**Figure 10F**).

FOS was associated with complement and coagulation cascades, Fc-gamma receptor (FcγR)-mediated phagocytosis, primary immunodeficiency, ribosome, spliceosome, and Toll-like receptor signaling pathway (**Figure 10G**).

MMP9 participated in antigen processing and presentation, complement and coagulation cascades, primary immunodeficiency, ribosome, RIG-I-like receptor signaling pathway, and Toll-like receptor signaling pathway (**Figure 10H**).

FOXO1 was involved in complement and coagulation cascades, hematopoietic cell lineage, ribosome, RIG-I-like receptor signaling pathway, T-cell receptor signaling pathway, and Toll-like receptor signaling pathway (**Figure 10I**).

STAT1 participated in ECM-receptor interaction, lysosome, neuroactive ligand-receptor interaction, olfactory transduction, proteasome, and spliceosome (**Figure 10J**).

Collectively, these results suggest that the hub genes are involved in diverse biological pathways related to immune response, signal transduction, metabolism, and cellular stress, highlighting their potential roles in the pathogenesis of HIV.

### 3.8 The correlation of hub genes and immune cell infiltration

**Figure 11:**
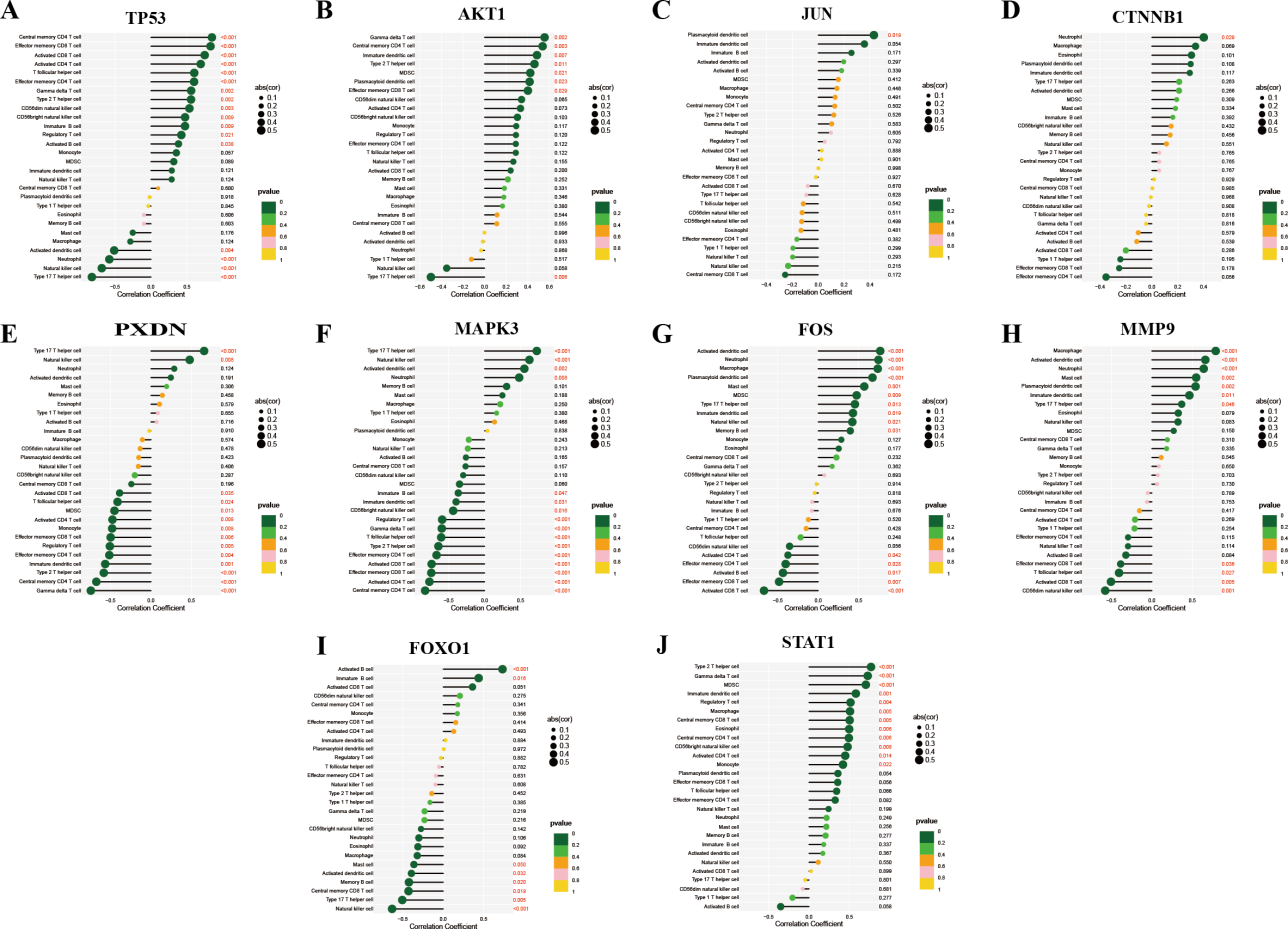
Correlations between hub genes and immune cells in HIV-positive patients. **(A–J)** show the correlations of TP53, AKT1, JUN, CTNNB1, PXDN, MAPK3, FOS, MMP9, FOXO1, and STAT1 with immune cell components, respectively. *P* < 0.05 was highlighted.

Considering the immune-related nature of HIV infection, we next examined correlations between hub gene expression and immune cell infiltration. TP53 was significantly positively correlated with central memory CD4+ T cells, effector memory CD8+ T cells, and activated CD8+ T cells, but negatively correlated with type 17 T helper cells, natural killer cells, neutrophils, and activated dendritic cells (**Figure 11A**). AKT1 showed significant positive correlations with gamma delta T cells, central memory CD4+ T cells, immature dendritic cells, type 2 T helper cells, MDSCs, plasmacytoid dendritic cells, and effector memory CD8+ T cells, whereas a negative correlation was observed with type 17 T helper cells (**Figure 11B**). JUN was significantly positively correlated with plasmacytoid dendritic cells (**Figure 11C**), while CTNNB1 was significantly positively correlated with neutrophils (**Figure 11D**). PXDN exhibited positive relationships with type 17 T helper cells and natural killer cells, but negative correlations with gamma delta T cells, central memory CD4+ T cells, type 2 T helper cells, and others (**Figure 11E**). MAPK3 demonstrated positive correlations with type 17 T helper cells, natural killer cells, activated dendritic cells, and neutrophils (**Figure 11F**), while it was negatively correlated with central memory CD4+ T cells, activated CD4+ T cells, and effector memory CD8+ T cells. FOS was significantly positively correlated with activated dendritic cells, neutrophils, macrophages, plasmacytoid dendritic cells, mast cells, MDSCs, type 17 T helper cells, immature dendritic cells, natural killer cells, and memory B cells, but negatively correlated with activated CD8+ T cells, effector memory CD8+ T cells, activated B cells, effector memory CD4+ T cells, and activated CD4+ T cells (**Figure 11G**). Macrophages, activated dendritic cells, and neutrophils were positively correlated with MMP9, while CD56dim natural killer cells, activated CD8+ T cells, T follicular helper cells, and effector memory CD8+ T cells exhibited negative correlations (**Figure 11H**). FOXO1 had significant positive correlations with activated B cells and immature B cells, while it showed negative correlations with natural killer cells, type 17 T helper cells, central memory CD8+ T cells, memory B cells, activated dendritic cells, and mast cells (**Figure 11I**). Finally, STAT1 demonstrated positive correlations with type 2 T helper cells, gamma delta T cells, and MDSCs (**Figure 11J**). These findings underscore the critical role of immune function in the pathogenesis and progression of HIV infection.

### 3.9 Drug-gene interaction network analysis

**Figure 12:**
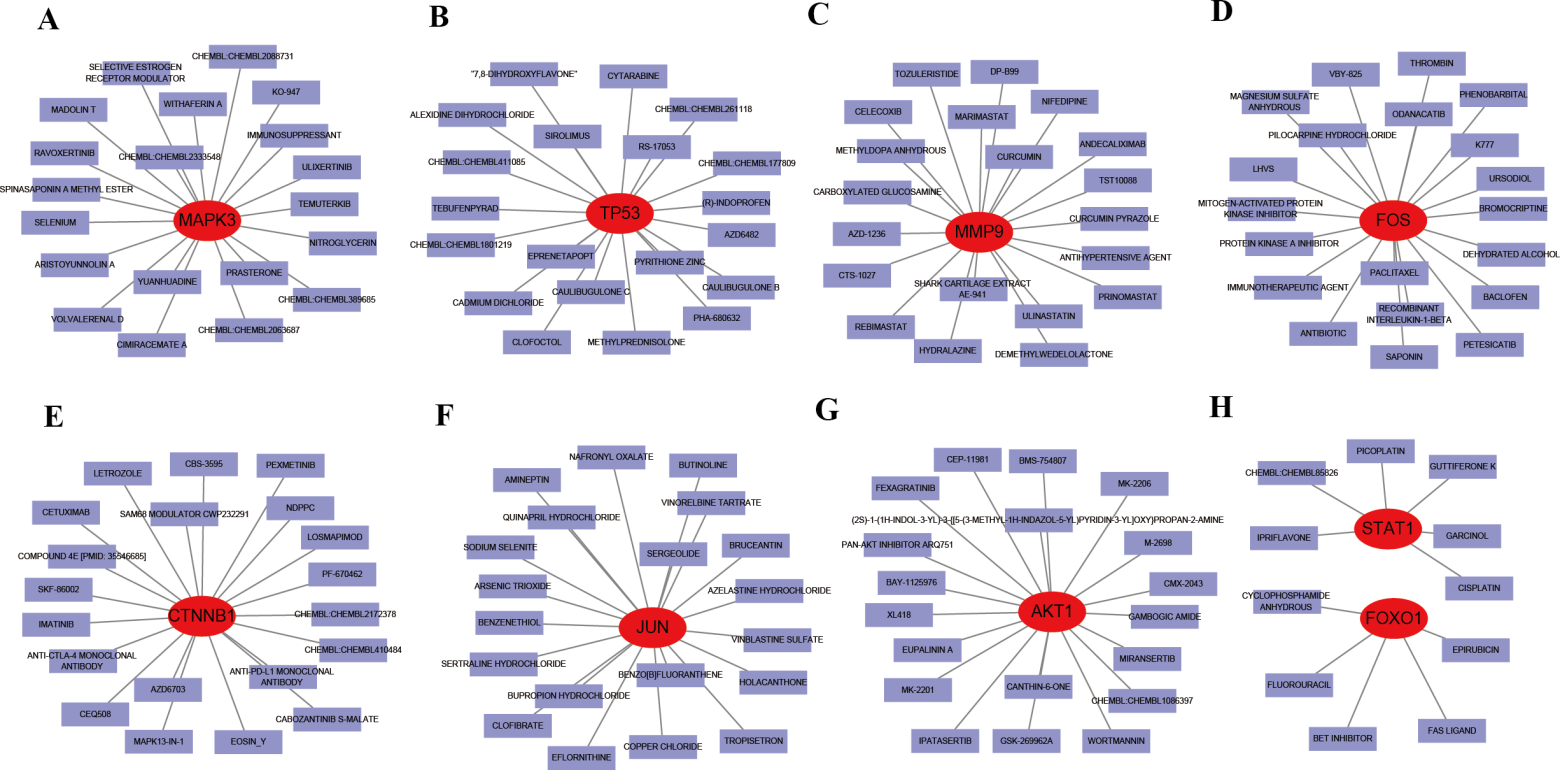
Interaction between existing therapeutic drugs and hub genes. **(A)** MAPK3. **(B)** TP53. **(C)** MMP9. **(D)** FOS. **(E)** CTNNB1. **(F)** JUN. **(G)** AKT1. **(H)** STAT1 and FOXO1.

Given the therapeutic implications of these hub genes, we explored their interactions with known drugs. MAPK3, TP53, MMP9, FOS, CTNNB1, and JUN were predicted to interact with 20 drugs each (**Figures 12A-F**), while AKT1 was predicted to interact with 19 drugs (**Figure 12G**). STAT1 and FOXO1 were predicted to interact with 6 and 5 drugs, respectively (**Figure 12H**). These drugs include CISPLATIN, PILOCARPINE HYDROCHLORIDE, CETUXIMAB, IMMUNOSUPPRESSANTS, and EPIRUBICIN, among others. Additionally, the drug-gene interactions were visualized using Cytoscape.

### 3.10 Construction of miRNA network

**Figure 13:**
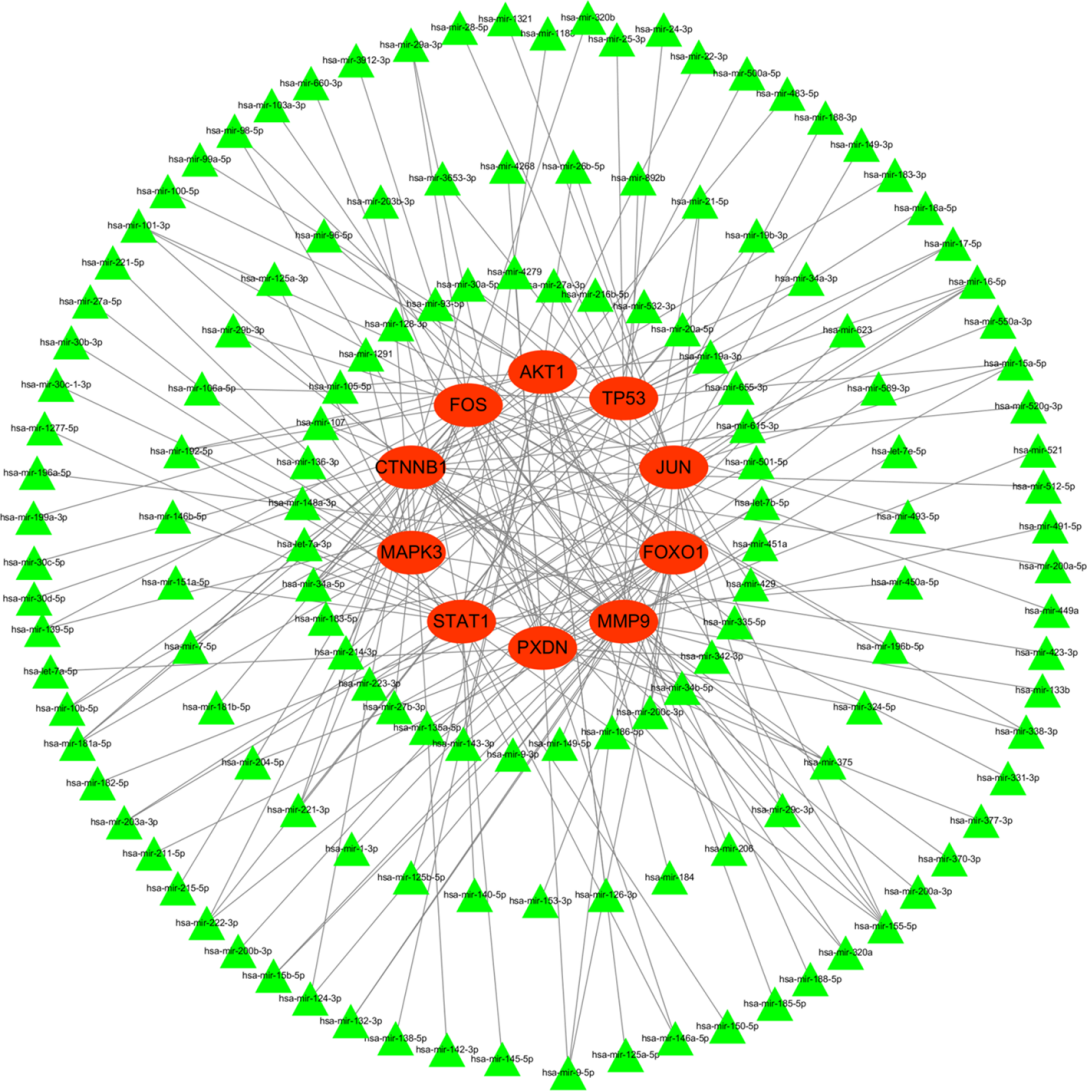
MiRNA-hub gene interaction networks.

To better understand post-transcriptional regulation, we constructed a miRNA–mRNA interaction network. **Figure 13** illustrated the interaction of hub genes (mRNAs) and their targeted miRNAs. The green triangle represents miRNA, while the red circle represents mRNA. All hub genes were regulated by multiple different miRNAs, and several miRNAs were found to regulate more than one hub gene.

### 3.11 Construction of transcriptional factor regulatory network

**Figure 14:**
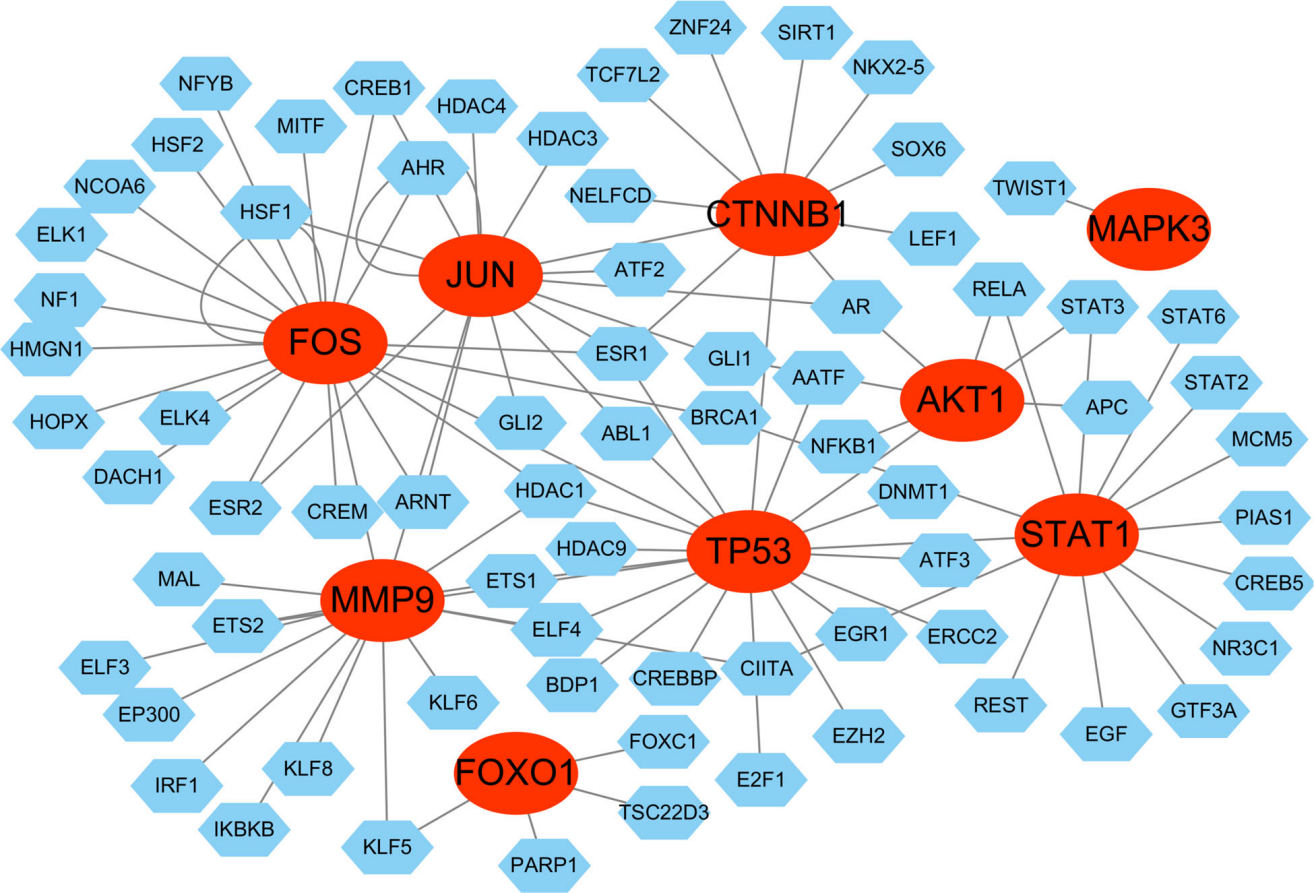
Transcriptional factor (TF) regulatory network. The red ovals represent hub genes, the blue hexagons represent transcriptional factors.

For the genes we identified, a gene-TF regulatory network was constructed including 101 interaction pairs among 9 hub genes and 72 TFs (**Figure 14**). While AKT1 was found to be regulated by 7 TFs, CTNNB1 by 10 TFs, FOS by 19 TFs, FOXO1 by 4 TFs, JUN by 14 TFs, MAPK3 by 1 TFs, MMP9 by 15 TFs, STAT1 by 14 TFs, and TP53 by 17 TFs. In addition, various TFs were found to regulate more than one hub gene, there are five transcriptional factors regulating three and more hub genes. For example, ESR1 was predicted to regulate CTNNB1, FOS, JUN and TP53; AR was found to regulate AKT1, CTNNB1 and JUN; FOS was found to regulate FOS, MMP9, and TP53. Specially, genes what transcriptional factors FOS and JUN regulated were themselves. Overall, these findings reveal a complex transcriptional regulatory network, with several key TFs, such as ESR1, AR, FOS, and JUN, exerting broad regulatory influence across multiple hub genes, including self-regulation.

## 4 Discussion

HIV infection remains incurable, with the primary barrier to eradication being the persistence of latent infection, particularly in memory CD4+ T cells (19). Although ART effectively suppresses viral replication, it fails to eliminate the virus completely. Consequently, long-term ART-treated HIV patients remain at risk of immune activation, chronic inflammation, and associated comorbidities (20), ultimately leading to increased mortality. Central to unraveling this conundrum is the regulation of gene expression, which is anticipated to play a pivotal role in the pathophysiology of HIV infection and its development. Therefore, it is essential to investigate the molecular markers and potential molecular pathways of HIV infection for the early detection and treatment, obviously, bioinformatic methods are promising methods. In the present study, 101 intersection genes were identified through Venn analysis by integrating DEGs, oxidative stress-related genes, and module genes obtained via WGCNA. A PPI network of these intersection genes was successfully constructed using the STRING online database and visualized with Cytoscape software. Finally, the top 10 hub genes (TP53, AKT1, JUN, CTNNB1, PXDN, MAPK3, FOS, MMP9, FOXO1, and STAT1) were identified from the PPI network using the CytoHubba plug-in in Cytoscape.

Among the ten identified hub genes, AKT1, MMP9, STAT1, and TP53 were significantly upregulated, whereas CTNNB1, FOS, FOXO1, JUN, MAPK3, and PXDN were downregulated in HIV-positive patients. The high diagnostic performance of these genes was demonstrated by ROC curve analysis. Notably, several genes, such as AKT1, TP53, and FOS, achieved an area under the curve (AUC) of 1.000, indicating their strong discriminatory ability between HIV-positive and HIV-negative individuals. Single-gene GSEA analysis revealed that the 10 hub genes were primarily enriched in core pathways including antigen processing and presentation (TP53, AKT1, PXDN, MAPK3, MMP9), complement and coagulation cascades (TP53, AKT1, JUN, PXDN, FOS, MMP9, FOXO1), Toll-like receptor signaling pathway (FOS, MMP9, FOXO1), and RIG-I-like receptor signaling pathway (FOXO1, MMP9). The antigen processing and presentation pathway, which mediates the recognition of viral antigens by MHC molecules, is a critical component of HIV-specific immune responses (21). Abnormal activation of the complement and coagulation cascades is closely associated with chronic inflammation and immune dysregulation caused by HIV infection, and such abnormal activation may contribute to immune pathological damage. The Toll-like receptor signaling pathway regulates innate immune responses through pattern recognition receptors, participating in viral recognition and the release of inflammatory factors. The enrichment of the RIG-I-like receptor signaling pathway further suggests the potential role of antibody-dependent cellular cytotoxicity and innate immune recognition in HIV control. These pathways collectively form the molecular network of HIV immune evasion, viral latency, and immune pathological damage, providing key targets for targeted intervention. Our further results indicate that these genes may play crucial roles in the pathogenesis of HIV infection by modulating key biological pathways, immune responses, and regulatory networks, including transcription factors, miRNAs, and drug-gene interactions.

TP53 is a well-established tumor suppressor gene involved in cell cycle regulation, DNA repair, senescence, autophagy, and apoptosis (22). The role of TP53 has been extensively studied in cancer and has also been implicated in HIV infection and its related pathogenesis, with multiple studies highlighting its involvement in immune activation, apoptosis, and the regulation of viral replication. Our analysis identified TP53 as a hub gene in HIV infection, showing a strong positive correlation with central memory CD4+ T cells, effector memory CD8+ T cells, and activated CD8+ T cells. This suggests that TP53 may play a role in T-cell-mediated immune responses during HIV infection. Previous studies have reported that TP53 promotes HIV long-terminal repeat (LTR) transcription and contributes to virus-induced cell death (23), highlighting its dual role in enhancing viral replication and mediating host cell apoptosis. In addition, TP53 interacts with the HIV-1 viral infectivity factor, thereby inducing G2 cell cycle arrest and promoting viral replication (24). Moreover, TP53 and its downstream gene p21 interfere with the early stage of HIV replication in non-cycling cells and human monocyte-derived macrophages (hMDMs) (14). Notably, research by Shin et al. (25) suggests that activation of the TP53 pathway enhances apoptosis in cells harboring latent HIV infection upon treatment with anticancer drugs, potentially offering a strategy to selectively eliminate HIV reservoirs. Except these functional insights, the TP53 R72P polymorphism has been investigated for potential epistatic effects in the context of HIV infection in human cohorts, although current findings have not demonstrated any statistically significant interactions or associations (26). Our findings highlight TP53 as a potential key player in HIV-related immune modulation and suggest that further investigation is warranted to explore its therapeutic implications.

AKT1 is a serine/threonine kinase involved in cell survival, immune regulation, and apoptosis. While it has been extensively studied in cancer and metabolic disorders (27–29). its role in infectious diseases, particularly HIV infection, remains an important research focus. Our analysis identified AKT1 as a hub gene in HIV infection, highlighting its involvement in viral transcription and transactivation through the PI3K/Akt and ROS/Akt signaling pathways (30, 31). Additionally, AKT1 participates in HIV infection via phosphorylation mechanisms (32). Notably, AKT1 has been proposed as a potential biomarker for distinguishing elite controllers from HIV+ patients and healthy individuals (8). Furthermore, its association with 19 therapeutic drugs suggests that AKT1 may serve as a key target for HIV-related interventions.

JUN and FOS are oncogenic transcription factors that regulate gene expression through the AP-1 transcription factor complex. They play essential roles in immune modulation, inflammation, and infection response. Our analysis identified JUN and FOS as hub genes in HIV infection, implicating their regulatory functions in viral latency and reactivation. JUN is involved in immune cell differentiation and inflammatory responses, which are critical in HIV pathogenesis. Notably, JUN functions at multiple stages of HSV-1 latent infection to promote reactivation (33), a mechanism that may be relevant to HIV latency. Transcriptional activation of JUN is also a hallmark of immune aging, contributing to inflammaging (34), which could influence HIV-related immune dysfunction. Beyond these roles, JUN is essential for diversification, function, and maintenance of identity of CD8+ T cells and cDC1 (35), it also enhances the cytotoxic activity of natural killer cells (36) and mediates the migration and differentiation of CD4+ T cells (37), all of which are crucial to the immune response during HIV infection. By influencing these immune cells, JUN plays a central role in the complex immune landscape of HIV infection, contributing to both immune evasion and dysfunction. Similarly, FOS, a proto-oncogene, participates in transcriptional regulation during HIV infection. As early as 1999, c-FOS was found to synergistically transactivate the HIV-1 long terminal repeat (LTR) via a MAPK-dependent pathway (38). Recent findings reveal that both JUN and FOS are upregulated in HIV DNA+ infected cells under suppressive therapy, suggesting their role in viral persistence and reactivation (39). Inhibition of c-FOS has been shown to enhance HIV-1 reactivation in latently infected cells, highlighting its potential as a therapeutic target. Interestingly, regulatory network analysis suggests that JUN and FOS form an autoregulatory feedback loop, which may influence gene expression dynamics during HIV infection.

In our study, CTNNB1, which encodes β-catenin, a core component of the canonical Wnt/β-catenin pathway involved in cell proliferation and differentiation (40), was found to be downregulated in HIV-positive patients. While CTNNB1 has been extensively studied in various cancers, such as endometrial and breast cancer, its role in infectious diseases, including HIV, remains unclear, with limited evidence suggesting no association with HBV infection (41). Similarly, PXDN, previously reported as a pan-cancer biomarker and linked to ocular disorders, was also downregulated in our analysis. However, the specific functions of CTNNB1 and PXDN in HIV infection are yet to be elucidated and warrant further investigation.

MAPK3, a key member of the MAPK family, is involved in multiple cellular processes and disease mechanisms, primarily through microRNA regulation and signaling pathways (42). In HIV infection, MAPK3 downregulation is associated with impaired activation of dendritic and natural killer cells, potentially contributing to immune evasion. However, our analysis did not reveal significant findings regarding MAPK3 in HIV patients and elite controllers (8). Notably, targeted inhibition of MAPK3 has demonstrated therapeutic benefits in various diseases. Our study predicts that drugs such as cisplatin, pilocarpine hydrochloride, cetuximab, and epirubicin may target hub genes including MAPK3, TP53, MMP9, and FOS, suggesting a potential therapeutic strategy for immune dysfunction-related diseases, including HIV infection.

MMP9 activity is known to increase with oxidative stress (43) and is upregulated by HIV-1 Tat via the MAPK-NF-kappaB signaling pathway (44). In our study, MMP9 expression was positively correlated with neutrophils, which aligns with immune infiltration data. Interestingly, we observed significantly lower levels of both neutrophils and MMP9 in TB/HIV co-infection compared to new pulmonary TB, likely due to the impaired production of neutrophils resulting from low CD4+ T-lymphocyte count and viability in co-infected individuals (45). Similarly, MMP9 plasma levels were higher in the treated HIV subgroup compared to the naïve subgroup (46), suggesting that HIV infection contributes to the reduced expression of MMP9. However, the expression of MMP9 in HIV patients remains controversial, with some studies showing high expression (47), while others found no impact from maternal HIV (48). A study on HIV-HCV co-infection revealed that ribavirin and interferon can alter MMP9 abundance *in vitro* and *in vivo* (49), highlighting the potential influence of drug-gene interactions in MMP9 regulation. FOXO1 is a key regulator of T-cell quiescence, FOXO1 activity promotes and maintains the quiescent state of memory CD4^+^ T cells, in which HIV establishes latency. In turn, FOXO1 inhibition reactivates HIV latent proviruses (19, 50). Additionally, the AKT/FOXO1 signaling axis plays a crucial role in HIV Tat protein accumulation (51). Moreover, FOXO1, targeted by LncRNA-GM, promotes Th17 differentiation while inhibiting Treg generation, exacerbating autoimmune inflammation (52). These findings highlight FOXO1 as a potential target for HIV latency reversal and immune modulation. STAT1 mainly exerts inflammatory biological functions. Additionally, we found that STAT1 was positively correlated with type 2 T helper cells and gamma delta T cells, both of which are essential for antiviral immunity. As one of the members of the IFN-γ-Stat1-T-bet axis involved in HIV infection, STAT1 play a key role in blunting Th1 response and suppressing CD4+ T cell activation (53). Along the same line, a report showed that higher STAT1 levels in CD4+ T cells are also associated with lower CD4+ T cell counts in HIV patients (54). Moreover, STAT1 signaling is involved in HIV-induced blood-brain barrier damage, which is relevant to viral neuropathogenesis (55). Given that HIV infection induces oxidative stress and immune dysregulation, targeting genes like MMP9, FOXO1, and STAT1 with specific inhibitors or modulators may help restore immune homeostasis and reduce HIV-related complications.

KEGG enrichment analysis revealed that these hub genes are involved in critical signaling pathways relevant to HIV infection, including antigen processing and presentation, complement and coagulation cascades, Toll-like receptor signaling, and apoptosis regulation. Specifically, AKT1, TP53, and STAT1 were implicated in antigen processing and presentation, a key mechanism in viral recognition and immune response activation. The involvement of JUN, FOXO1, and MAPK3 in complement and coagulation cascades suggests their potential role in immune-mediated inflammation and coagulopathy, which are frequently observed in HIV-infected individuals. Notably, AKT1, FOXO1, and MMP9 were enriched in the RIG-I-like receptor and Toll-like receptor pathways, which are crucial for innate immune responses against viral infections. These findings highlight the potential of these hub genes as therapeutic targets for modulating immune responses in HIV infection.

In our study, we further explored the post-transcriptional regulatory landscape of the identified hub genes by predicting their corresponding miRNAs. The construction of a miRNA–mRNA regulatory network revealed that several miRNAs may play central roles in modulating hub gene expression during HIV infection. Notably, miR-34a-5p and miR-1-3p were predicted to target all ten hub genes, indicating their potential involvement in broad regulatory networks. The identification of these interactions highlights the importance of miRNA-mediated regulation in shaping the immune response and cellular environment in HIV-positive individuals. Some of the predicted miRNAs have previously been implicated in viral replication, immune evasion, or chronic inflammation. For instance, Zhang et al. (56) proposed that the HIV Tat protein induces the overexpression of miR-34a in TZM-bl cells through transactivation of the LTR via the SIRT-1/NF-κB pathway. The upregulation of miR-34a, in turn, enhances viral transactivation by affecting Tat expression and increasing its activity. Additionally, Kapoor et al. (57) reported that miR-34a is upregulated in T cells during HIV infection. They further indicated that miR-34a enhances HIV replication in T cells by overcoming the inhibitory effect of the host protein phosphatase 1 nuclear targeting subunit (PNUTS), which aligns with the biological functions of hub genes like TP53 and AKT1.

Finally, our discussion should focus on the potential therapeutic implications of the identified hub genes and pathways. The hub genes identified in this study—TP53, AKT1, JUN, CTNNB1, PXDN, MAPK3, FOS, MMP9, FOXO1, and STAT1—play pivotal roles in various immune regulatory processes that are dysregulated in HIV infection. Given their central role in both HIV pathogenesis and immune dysregulation, these genes and their associated signaling pathways present promising targets for therapeutic intervention. Several strategies could be employed to modulate these genes and mitigate HIV-induced immune dysfunction. One promising approach involves the use of small molecule inhibitors to target key signaling pathways, such as those involving TP53, AKT1, and MAPK3. For instance, AKT inhibitors, which have already been explored in other cancers and chronic diseases, could potentially help restore normal T cell function and prevent apoptosis of immune cells in HIV-infected patients. Furthermore, targeting the MAPK3 pathway could help reduce inflammation and control immune responses, which are often hyperactivated in HIV. Another viable strategy is the application of monoclonal antibodies (mAbs) directed against immune checkpoint proteins or pro-inflammatory signaling mediators. For example, monoclonal antibodies against FOS or STAT1 could help modulate the immune response, particularly in reducing the activation of inflammatory pathways that contribute to HIV-induced immune dysregulation. Similarly, targeting JUN could offer a strategy to interfere with the transcriptional regulation of pro-inflammatory genes, thus mitigating chronic inflammation associated with HIV infection. Gene editing technologies, such as CRISPR-Cas9, offer yet another avenue for intervention. Upregulation of beneficial genes like TP53 or suppression of genes such as JUN and STAT1 could correct immune imbalances and reduce HIV-associated inflammation. These approaches may enable precision-targeted modulation of dysfunctional immune pathways, offering a personalized therapeutic platform. Furthermore, since several hub genes, such as PXDN, FOS, and MAPK3, are involved in oxidative stress and apoptotic signaling pathways, targeting oxidative stress pathways with antioxidant therapies or small molecule inhibitors could reduce oxidative damage to immune cells and limit HIV-induced apoptosis. This approach may help prevent the early loss of immune cells and reduce HIV-associated immune dysfunction. Given the complex interactions between the identified hub genes and the immune system, combination therapies that target multiple pathways simultaneously may prove to be the most effective. For instance, combining inhibitors of AKT1 and TP53 with monoclonal antibodies targeting immune checkpoints could synergistically enhance the immune response and prevent HIV latency, which is a significant hurdle in the treatment of HIV infection. In conclusion, targeting these hub genes through small molecule inhibitors, monoclonal antibodies, gene editing, and modulation of oxidative stress pathways represents a promising avenue for therapeutic intervention in HIV-positive patients.

Admittedly, the limitations of this study should also not be ignored. Firstly, the dataset (GSE76246) lacks specific data on CD4+T cell counts and cannot be used for clinical staging studies based on the immune status of HIV patients. Secondly, although promising results were obtained through this bioinformatics analysis, the functions and mechanisms of these hub genes remain experimentally unvalidated, which could lay the groundwork for a deeper understanding of HIV infection. Thirdly, a much larger sample size is needed to avoid a selective bias. Therefore, verifying the functions of these hub genes by experimental and molecular studies may be an area of future research. Last but not least, the development of targeted therapies aimed at restoring immune balance and reducing HIV-associated complications should serve as the ultimate goal.

## 5 Conclusion

In summary, through comprehensive bioinformatics analysis, TP53, AKT1, JUN, CTNNB1, PXDN, MAPK3, FOS, MMP9, FOXO1, and STAT1 were identified and confirmed as hub genes, which may play key roles in the pathogenesis of HIV infection.

## Data Availability

The datasets presented in this study can be found in online repositories. The names of the repository/repositories and accession number(s) can be found in the article/supplementary material.
